# The efficacy of a thrombin-based hemostatic agent in primary total knee arthroplasty: a meta-analysis

**DOI:** 10.1186/s13018-014-0090-7

**Published:** 2014-10-15

**Authors:** Chen Wang, Zhe Han, Tao Zhang, Jian-xiong Ma, Xuan Jiang, Ying Wang, Xin-long Ma

**Affiliations:** Biomechanics Labs of Orthopaedics Institute, Tianjin Hospital, Tianjin, 300050 People’s Republic of China; Tianjin Medical University, Tianjin, 300070 People’s Republic of China; Department of Orthopedics, Tianjin Hospital, Tianjin, 300211 People’s Republic of China; General Hospital of Tianjin Medical University, Tianjin, 300052 People’s Republic of China

**Keywords:** Floseal®, Thrombin, Arthroplasty, Meta-analysis

## Abstract

**Purpose:**

Total knee arthroplasty (TKA) is a popular procedure in severe osteoarthritis. But perioperative bleeding remains a problem. Floseal® is a mixture of thrombin and bovine gelatin which can benefit a lot on reducing intraoperative and postoperative bleeding. However, there is no enough evidence judging its safety and efficiency. So a meta-analysis is conducted by us to evaluate the efficacy and safety of a thrombin-based hemostatic agent compared with conventional methods in TKA.

**Method:**

Two independent reviewers selected literatures published before August 2014 from MEDLINE, Embase, and The Cochrane Central Register of Controlled Trials. Other internet databases were also performed to identify trials according to the Cochrane Collaboration guidelines. High-quality randomized controlled trials (RCTs), prospective control trials (PCTs), and case controlled trials (CCTs) were selected. The meta-analysis was undertaken using RevMan 5.1 for Windows.

**Results:**

Three RCTs, one PCT, and one CCT met the inclusion criteria. There were significant differences in hemoglobin decline and calculated total blood loss between the Floseal® group and control group. There were no significant differences in postoperative drainage volume, rate of transfusion requirement, incidence of wound infection, deep vein thrombosis (DVT), and pulmonary embolism (PE) between treatment and control groups.

**Conclusions:**

The present meta-analysis indicates that a thrombin-based hemostatic agent can reduce hemoglobin decline and calculated total blood loss after TKA and is not related to adverse reactions or complications such as wound infection, DVT, and PE.

## Introduction

Total knee arthroplasty (TKA) has become a common treatment for severe knee arthritis [1]. However, because of extensive soft tissue dissection, long operative time, and a large amount of bone cutting, patients undergoing primary TKA are particularly prone to large intraoperative and postoperative blood loss which may lead to morbidity including pain, decrease range of motion, and delayed recovery. Although various methods have been taken by surgeons to reduce blood loss including electrocoagulation, cell salvage, predonated autologous blood transfusion, hemostatic agents, erythropoietic agents, and minimally invasive surgery, many patients still need blood transfusion for significant blood loss [2]. Besides, fibrinolysis reaction activated by surgical injury and ischemia reperfusion after tourniquet deflation contribute to the blood loss [3,4].

Allogenic blood transfusion may be related to systemic complications such as anaphylactic reaction, immunological rejection, hemolytic reaction, infectious disease, and metabolic disorders prolonging the duration of hospitalization and causing serious social economic burden [5]. So it is crucial to minimize the bleeding and transfusion requirements for the patients undergoing TKA.

Hemostatic agent Floseal® is a mixture of thrombin and bovine gelatin [6] which can benefit a lot on reducing intraoperative and postoperative bleeding and transfusion requirements. Thrombin-based hemostatic agents have come out nearly 15 years and were widely used in surgical procedure including gynecology, general surgery, and orthopedics which were still attracting the attention and interest of multitudinous surgeons. Clapp M [7] found Floseal® can achieve hemostasis during a laparoscopic salpingotomy and preserve tubal patency. Testini M [8] considered that Floseal® was an effective additional agent compared with conventional hemostatic procedures in thyroid surgery. A number of clinical trials have been made to evaluate the efficacy and safety of the hemostatic matrix in primary TKA. However, some have been criticized for poor designs, inaccurate evaluations, inconclusive results, and short-term follow-ups. Therefore, we conducted a meta-analysis, pooling the data from randomized controlled trials (RCTs) and non-RCTs to provide an evidence-based judgment regarding the use of a thrombin-based hemostatic agent in patients undergoing primary TKA.

## Methods

### Search strategy

**Figure 1:**
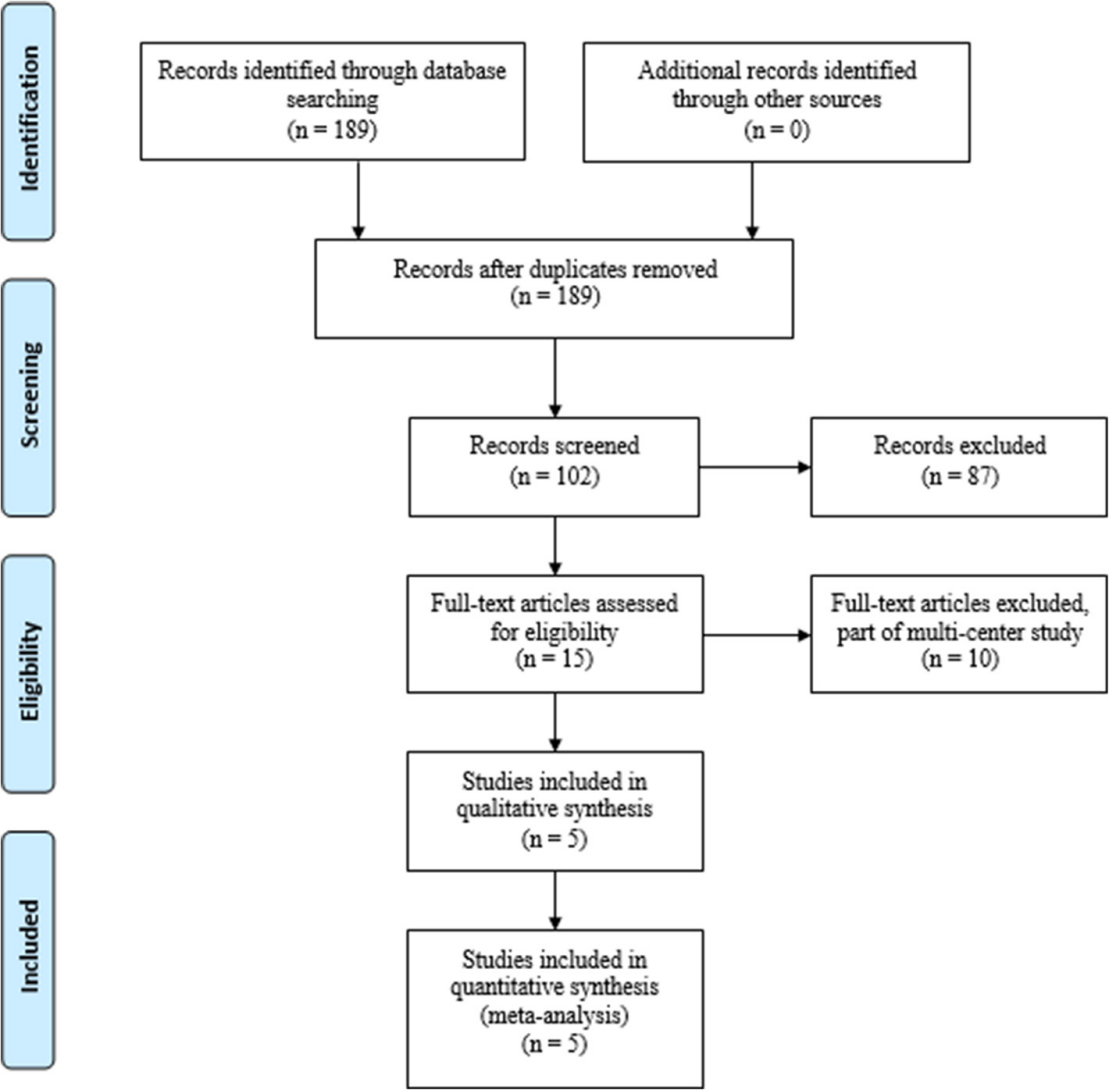
**Search results and the selection procedure.**

We conducted a meta-analysis to identify academic articles from electronic databases, including MEDLINE (1966 to August 2014), Embase (1980 to August 2014), and The Cochrane Central Register of Controlled Trials. Other internet databases were also performed to identify trials according to the Cochrane Collaboration guidelines. There were no language restrictions. The search strategy is presented in Figure 1. It included only studies conducted on human subjects. In addition, using the Google search engine, the same search terms were manually searched to find any further relevant studies that may have been missed in the database search. We used the following key words: “knee replacement OR arthroplasty” and “thrombin” in combination with Boolean operators AND or OR.

### Selection criteria and quality assessment

We included all published RCTs and non-RCTs comparing Floseal® with a control (placebo or nothing) in patients undergoing primary TKA. The methodological quality of the included studies was assessed by the review authors using a modification of the generic evaluation tool used by the Cochrane Bone, Joint and Muscle Trauma Group. To provide a qualification of a bias risk, quality criteria included (i) details of randomization method, (ii) allocation concealment, (iii) blinding of participants and personnel, (iv) blind outcome assessment, (v) incomplete outcome data, (vi) selective outcome reporting, and (vii) other sources of bias.

### Data extraction

For each eligible study, two of the authors independently extracted all the relevant data. Disagreement was resolved by a discussion with the third reviewer. Whenever necessary, we contacted the authors of the studies for missing data or further information. The following data were extracted: (1) demographic data of participants; (2) indication for TKA; (3) wound infection (superficial or deep), hematoma, wound dehiscence, limb swelling, bleeding from the wound, and reoperation because of a wound-healing complication; (4) postoperative blood transfusion, decrease in hemoglobin or hematocrit, thromboembolic complications, patient discomfort, and costs; (5) functional outcomes such as time to regain mobility; and (6) any other outcomes as mentioned in individual studies were considered for inclusion. In studies in which data were incomplete or unclear, attempts were made to contact investigators for clarification.

### Data analysis and statistical methods

The meta-analysis was undertaken using RevMan 5.1 for Windows (The Cochrane Collaboration, Oxford, United Kingdom). We assessed statistical heterogeneity for each study with the use of a standard chi-square test (for heterogeneity, a level of *P* < 0.1 was considered significant) and the *I*^2^ statistic. An *I*^2^ statistic value of 50% was considered to indicate substantial heterogeneity. The origins of heterogeneity, if present, were analyzed according to differences in methodological quality, characteristics of participants, and intervention. When the data allowed, the authors of this paper performed subgroup analysis of the trials. In comparing trials showing heterogeneity, pooled data were meta-analyzed using a random-effects model. Otherwise, a fixed-effects model was used for the analysis. Relative risks (or risk differences) and 95% confidence intervals (CIs) were calculated for dichotomous outcomes and mean differences (MDs) and 95% CIs for continuous outcomes.

## Result

### Search result

We identified a total of 189 citations as potentially relevant. By screening the title and reading the abstract and the entire article, we found that three RCTs and two non-RCTs enrolling a total of 766 knees at a final follow-up were eligible for data extraction and meta-analysis [9–13]. The patients’ characteristics were presented in Table 1. The sample size for each study ranged from 20 to 349. Jadad scale is used to evaluate the quality of RCTs while MINORS assessment is used for non-RCTs. The quality of the literature is relatively high. But there are still some methodological limitations in RCTs. All the RCTs [9–11] reported that randomization was conducted through a computer-assisted program. A double-blind methodology was reported in two RCTs [10,11]. None of the RCTs suggest a methodology for the blinding assessors. All of the studies provided results for a minimum of 95% of the included patients. Most of the patients who managed to undergo primary TKA suffered from osteoarthritis. Kim and Comadoll [11,13] reported that all TKA were accomplished by the same doctor. Cemented prosthesis was used in four studies [10–13]. The other one used an uncemented prosthesis. Electrocautery hemostasis combined with a thrombin-based hemostatic agent was used in the treatment group in all the studies except one [12] which used an agent only. The control group received electrocautery hemostasis. Four literatures [9–12] mentioned volume of drainage in 24 h postoperatively. None of the studies reported whether drainage was clamped or not, while two studies [10,11] stated the size and number of the drainage tube. Tourniquets were used in all the inclusive patients and were deflated after wound closure in three studies [9–11] and before wound closure in Comadoll’s study. Helito did not provide this information while the pressure of the tourniquet was recorded (300 mmHg) [12]. Two studies [10,11] applied bandage after wound closure. Spinal anesthesia was performed in the study of Kim and Helito [11,12]. Others did not mention an anesthesia method. The dosage of Floseal® ranged from 10 to 20 ml, 10 ml in three studies [9–11] and 20 ml in Comadoll’s trial. None of the studies used placebo in a control group. Four studies [9,10,12,13] provided the indications of blood transfusion, which based on postoperative hemoglobin level, signs and symptoms of anemia such as hypotension, paleness, and tachycardia. Perioperative antithromboembolic prophylaxis therapy was performed in all the patients as followed in Table 1. The duration of a follow-up ranged from discharge to 6 weeks.

**Table 1:** **Cohort characteristics**

**Studies**	**Cases (F/C)**	**Mean age (F/C)**	**Male patient (F/C)**	**Dosage (ml)**	**Prophylactic antithrombotic**	**Reference type**	**Quality assessment score**
Kim 2012 [11]	97/99	72.7/70.1	NS	10	Aspirin or warfarin, NS	RCT	6
Francesco 2013 [9]	51/42	67.9/70.2	24/17	10	LMWH 4,000 IU	RCT	4
Suarez 2014 [10]	56/52	65.9/65.1	20/21	10	Enoxaparin, NS	RCT	6
Helito 2013 [12]	10/10	67.8/66.6	NS	NS	Enoxaparin 40 mg	PCT	17
Comadoll 2012 [13]	184/165	NS	64/54	20	Warfarin 5 mg	CCT	18

*F* Floseal®, *C* control, *LMWH* low molecular weight heparin, *NS* not stated.

### Meta-analysis results

#### Hemoglobin decline

**Figure 2:**
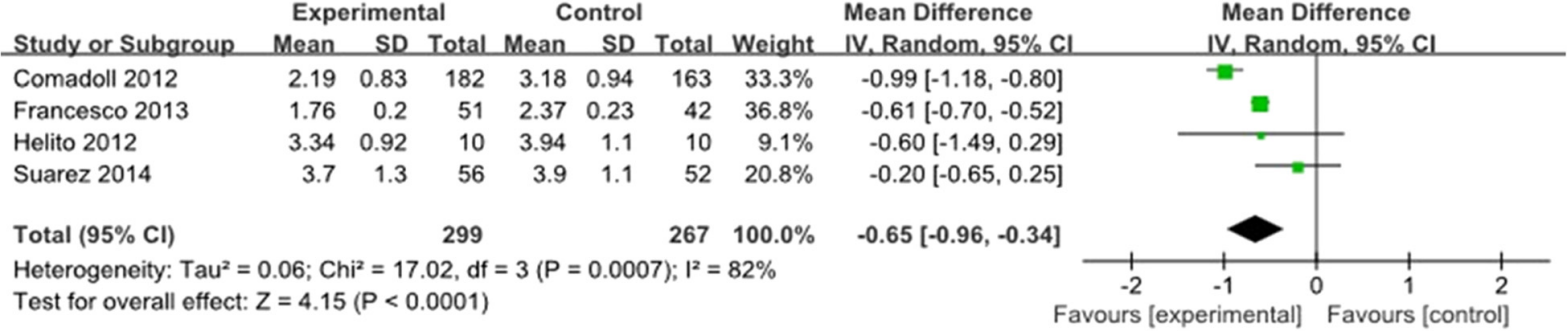
**Forest plot diagram showing the effect of Floseal® on hemoglobin drop.**

We obtained usable data on hemoglobin drop from four trials including 566 knees [9,10,12,13]. As depicted in Figure 2, there was significant heterogeneity (*χ*^2^ = 17.02, df = 3, *I*^2^ = 82%, *P* = 0.0007). Using a random-effects model, the pooled results indicated that there was significant difference between the groups in terms of hemoglobin drop (MD = −0.65, 95% CI: −0.96 to −0.34, *P* <0.0001). Subgroup analysis was performed for postoperative hemoglobin decline which showed that the existence of heterogeneity was due to the different doses of Floseal® and duration of drainage (Table 2).

**Table 2:** **Subgroup analysis of postoperative hemoglobin decline**

**Outcome or subgroup**	**Studies**	**Effect estimate**				
		*χ* ^2^	*I* ^2^ (%)	MD	95% CI	*P* value
Cemented prosthesis	3	10.31	81	−0.62	[−1.22,−0.03]	0.006
Total dose = 10 ml	3	3.03	34	−0.52	[−0.77,−0.26]	0.22
Duration of drainage	3	3.03	34	−0.52	[−0.77,−0.26]	0.22

#### Volume of drainage

**Figure 3:**
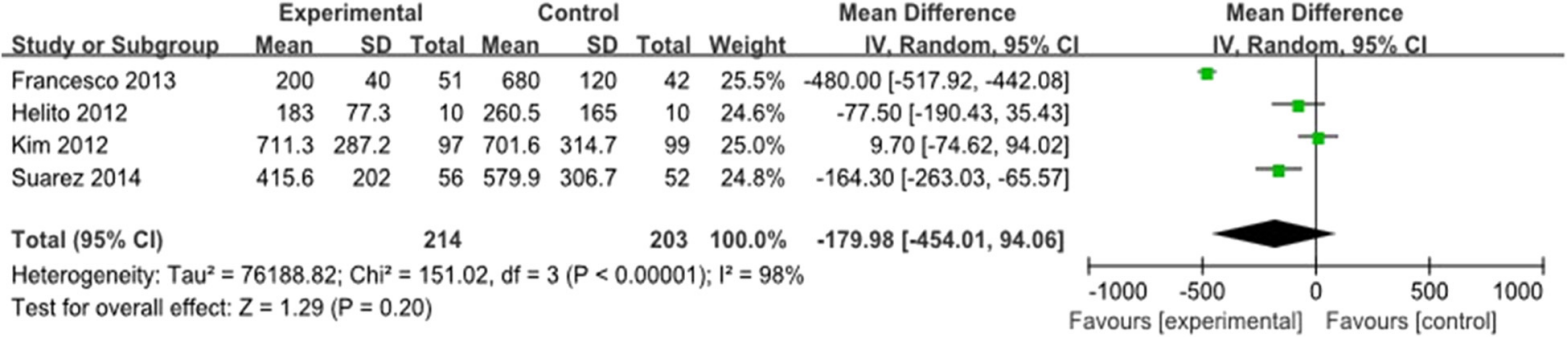
**Forest plot diagram showing the effect of Floseal® on drainage volume.**

The volume of drainage was mentioned in four trials [9–12]. The pooled results showed significant heterogeneity (*χ*^2^ = 151.02, df = 3, *I*^2^ = 95%, *P* < 0.00001; Figure 3). Thus, a random-effects model was used. Meta-analysis showed no significant difference between the groups in terms of volume of drainage (MD = −179.98, 95% CI: −454.01 to 94.06, *P* = 0.20).

#### Need for transfusion

**Figure 4:**
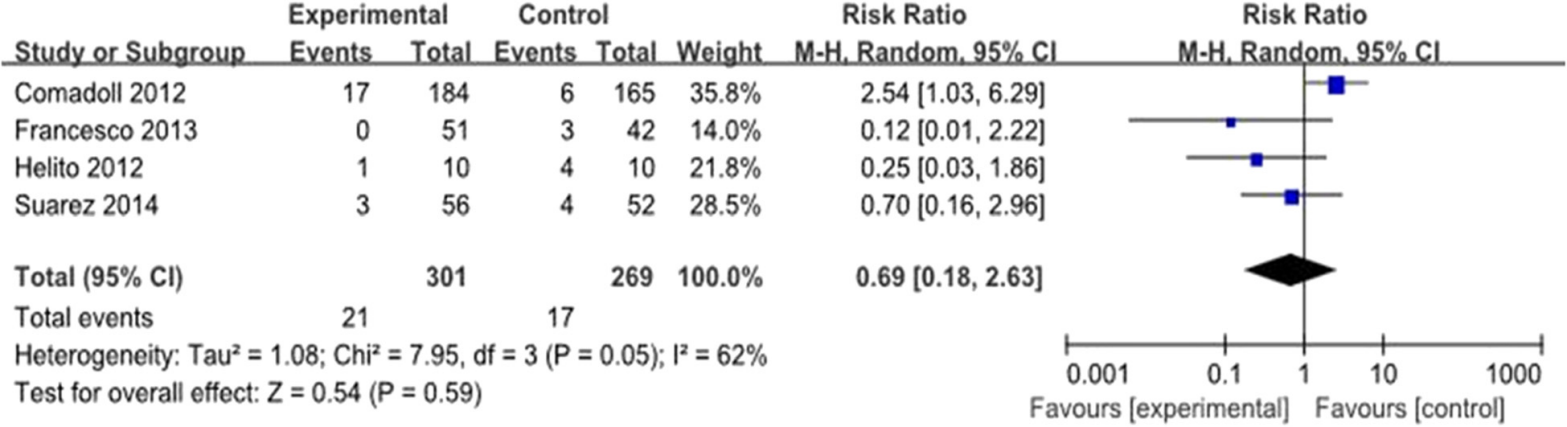
**Forest plot diagram showing the effect of Floseal® on drainage volume on the need for transfusion.**

The need for transfusion was reported in four trials [9,10,12,13]. The pooled results showed no significant difference between the groups (RR = 0.69, 95% CI: 0.18 to 2.63, *P* = 0.05). There was significant heterogeneity (*χ*^2^ = 7.95, df = 3, *I*^2^ = 62%, *P* =0.05; Figure 4). A random-effects model was used.

#### Total blood loss

**Figure 5:**
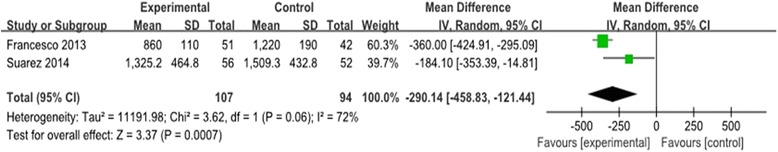
**Forest plot diagram showing the effect of Floseal® on drainage volume total blood loss.**

Total blood loss was documented in two studies [9,10]. The difference between the groups was significant (MD = −290.14, 95% CI: −458.83 to −121.44, *P* = 0.0007; Figure 5). A random-effects model was used because statistical heterogeneity was found between the studies (*χ*^2^ = 3.62, df = 1, *I*^2^ = 72%, *P* = 0.06).

#### Infection

**Figure 6:**
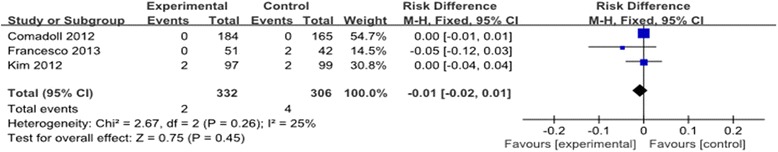
**Forest plot diagram showing the effect of Floseal® on the incidence of infection.**

The incidence of infection was reported in three studies [9,11,13]. The pooled results indicated that the incidence of infection was 0.6% in the Floseal® group, compared with 0.13% in the conventional group. This difference was not significant (RD = −0.01, 95% CI: −0.02 to 0.01, *P* = 0.45; Figure 6). A fixed-effects model was used because no statistical heterogeneity was found between the studies (*χ*^2^ = 2.67, df = 2, *I*^2^ = 25%, *P* = 0.26).

#### Deep vein thrombosis (DVT)

**Figure 7:**
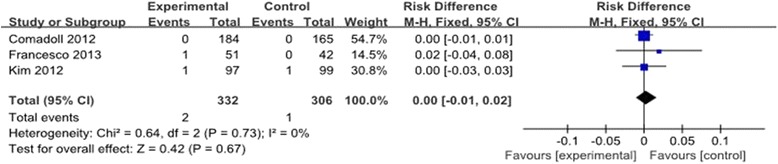
**Forest plot diagram showing the effect of Floseal® on the incidence of DVT.**

The incidence of DVT was reported in three trials [9,11,13]. The pooled results of the trials showed that the use of Floseal® did not increase the risk of DVT. (RD = 0.00, 95% CI: −0.01 to 0.02, *P* = 0.67). There was no significant heterogeneity (*χ*^2^ = 0.64, df = 2, *I*^2^ = 0%, *P* =0.73; Figure 7). A fixed-effects model was used.

#### Pulmonary embolism (PE)

**Figure 8:**
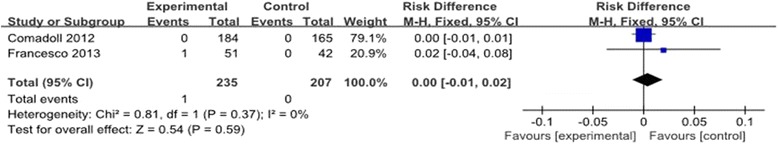
**Forest plot diagram showing the effect of Floseal® on the incidence of PE.**

The incidence of PE was mentioned in two trials [9,13]. The pooled results of the trials showed that the use of Floseal® did not increase the risk of DVT. (RD = 0.00, 95% CI: −0.01 to 0.02, *P* = 0. 59). There was no significant heterogeneity (*χ*^2^ = 0.81, df = 1, *I*^2^ = 0%, *P* =0.37; Figure 8). A fixed-effects model was used.

#### Other outcomes

Several other outcome measures were identified, but insufficient data were provided for meta-analysis. For instance, Suarez found no significant differences in overall length of stay, hematocrit values, and postoperative fluids or hidden blood loss between two study groups [10]. There were no significant differences in colloid and crystalloid infusion between two groups in the study of Helito [12].

## Discussion

The most important finding of the present meta-analysis was that the use of thrombin-based hemostatic agents can decrease the postoperative hemoglobin drop and total blood loss. Furthermore, no significant differences were found regarding to the risk of wound infection, DVT, and PE.

Three RCTs, one PCT, and one CCT met the inclusion criteria for the meta-analysis. All the RCTs stated that randomization was achieved by a computer-assisted program. The overall methodological quality of the included studies was relatively high. Two RCTs [10,11] reported a methodology of double-blind, and no studies were provided to blind the assessors of group allocation so expectation bias and the potential for type II statistical error in their clinical outcomes may affect the analysis. All included studies had consistent baseline data, but none of them mentioned intention to treat analysis. These methodological strengths and weaknesses should be considered when interpreting the findings of the present meta-analysis.

TKA is complicated by perioperative blood loss; 7.7%–18.93% of the patients undertaking TKA need transfusion [14–17]. The present meta-analysis indicated that there was no significant difference in the rate of transfusion between two groups (6.98% vs 6.32%). The reason could be an unmentioned transfusion trigger in the literature, different approach, and type of anesthesia in the surgical procedure. Besides various baselines of a preoperative hemoglobin level, different dosage and kinds of antithrombotic drugs between two groups also should be taken into consideration.

Four studies [9–12] mentioned postoperative volume of drainage. Our studies found no statistically significant differences between the treatment and control groups. Some literatures [12,13] did not provide the time of drainage discharge or the number of drainage tubes. What is more, varied methods of anesthesia and surgical approach may also affect the meta-analysis.

Anemia may lead to hypotension, cardiac failure, and even shock. Blood transfusion was needed to correct severe anemia. Four studies [9,10,12,13] reported a postoperative hemoglobin decline. Two literatures [9,10] mentioned total blood loss. The present meta-analysis showed significant differences in postoperative hemoglobin decline and total blood loss. Subgroup analysis showed the source heterogeneity for postoperative hemoglobin decline (Table 2). Extravasation into the tissues is the major hidden postoperative blood loss. While apparent postoperative blood loss can be recorded through drainage bottles. From a present meta-analysis, we consider that the use of thrombin-based hemostatic agents can reduce hemoglobin drop and perioperative blood loss.

Infection is relatively rare after TKA but can be devastating in terms of morbidity and cost [18]. This meta-analysis found no significant difference in the incidence of infection, which was 0.6% with treatments and 0.13% in controls; the overall infection rate was 0.94%. The reported incidence of infection after TKA ranged from 1% to 3% [19]. Because infection may also occur later, an assessment after a longer follow-up period may be required.

DVT is one of the common complications after TKA which may cause PE and even death [20]. Antithromboembolic prophylaxis therapy was adapted in all the inclusive patients, and there was no difference in the incidence of DVT (0.6% vs 0.33%) or PE (0.43% vs 0%) between the two groups.

There are several potential limitations of our meta-analysis. (1) Only five reports were included, and their sample sizes were relatively small, which may have affected our conclusions. (2) The follow-up of patients in some of the trials was unclear. Many patients were followed up in the short term. This may have resulted in underreporting of, for example, infection. (3) There were insufficient data to support analyses of length of stay, range of motion (ROM), postoperative fluids, and Visual Analog Scale Pain Score. (4) The existence of a publication bias also affects the analysis; it is a limitation in all meta-analysis. However, this is the first systematic review to evaluate safety and efficiency of thrombin-based hemostatic agents during TKA, by only including studies that have appropriate control and study groups. All of the included studies were of high quality. Further studies should be designed to explore an optimal dose and additional functions during the TKA based on high quality RCTs.

## Conclusion

The present meta-analysis indicates that thrombin-based hemostatic agents can reduce hemoglobin decline and total blood loss after TKA and are not related to the adverse reactions or complications such as wound infection, DVT, and PE. In summary, the use of thrombin-based hemostatic agents is superior to standard electrocautery in patients undergoing primary TKA.
